# Assessing the Influence of a Fitbit Physical Activity Monitor on the Exercise Practices of Emergency Medicine Residents: A Pilot Study

**DOI:** 10.2196/mhealth.6239

**Published:** 2017-01-31

**Authors:** Justin David Schrager, Philip Shayne, Sarah Wolf, Shamie Das, Rachel Elizabeth Patzer, Melissa White, Sheryl Heron

**Affiliations:** ^1^ Department of Emergency Medicine Emory University School of Medicine Atlanta, GA United States; ^2^ Department of Surgery Emory University School of Medicine Atlanta, GA United States

**Keywords:** activity trackers, personal fitness trackers, physical fitness trackers, medical residency, wellness programs, mobile health

## Abstract

**Background:**

Targeted interventions have improved physical activity and wellness of medical residents. However, no exercise interventions have focused on emergency medicine residents.

**Objective:**

This study aimed to measure the effectiveness of a wearable device for tracking physical activity on the exercise habits and wellness of this population, while also measuring barriers to adoption and continued use.

**Methods:**

This pre-post cohort study enrolled 30 emergency medicine residents. Study duration was 6 months. Statistical comparisons were conducted for the primary end point and secondary exercise end points with nonparametric tests. Descriptive statistics were provided for subjective responses.

**Results:**

The physical activity tracker did not increase the overall self-reported median number of days of physical activity per week within this population: baseline 2.5 days (interquartile range, IQR, 1.9) versus 2.8 days (IQR 1.5) at 1 month (*P*=.36). There was a significant increase in physical activity from baseline to 1 month among residents with median weekly physical activity level below that recommended by the Centers for Disease Control and Prevention at study start, that is, 1.5 days (IQR 0.9) versus 2.4 days (IQR 1.2; *P*=.04), to 2.0 days (IQR 2.0; *P*=.04) at 6 months. More than half (60%, 18/30) of participants reported a benefit to their overall wellness, and 53% (16/30) reported a benefit to their physical activity. Overall continued use of the device was 67% (20/30) at 1 month and 33% (10/30) at 6 months.

**Conclusions:**

The wearable physical activity tracker did not change the overall physical activity levels among this population of emergency medicine residents. However, there was an improvement in physical activity among the residents with the lowest preintervention physical activity. Subjective improvements in overall wellness and physical activity were noted among the entire study population.

## Introduction

Medical resident wellness, burnout, and lack of self-care is a multifaceted problem complicated by long work hours, demanding work environments, and a multitude of psychosocial stressors [1,2]. The recent suicides of 2 medical residents in New York [3] has refocused the conversation and has motivated leaders of medical training institutions to pilot interventions for improving resident wellness and decreasing burnout. Interventions to improve quality of life have included topics such as duty hour changes, stress reduction programs, interpersonal skill building, professional development, mentoring programs, physical activity, and psychotherapy [4-8]. Programs designed to improve physical fitness among residents and physicians have shown promise [7,9-11].

Medical residents have been found in multiple studies to have low levels of physical activity [4,12,13]. Specifically, internal medicine residents have been shown to have low levels of physical activity, with only 15% of them being above average or excellent [12]. In a national survey, resident physicians met the US Department of Health and Human Services (USDHHS) guidelines for physical activity approximately 73% of the time, but this percentage was lower than that for both attending physicians (84.8%) and medical students (84%). These results suggest that an intrinsic characteristic of life in residency training decreases a person’s physical activity levels [13,14]. Physical activity among physicians is not only important for their own health, well-being, and career longevity, but is also correlated with their individual practice of counseling their patients on the benefits of exercise [15-17].

The majority of wellness interventions have focused on internal medicine and surgery residents, while few have focused on emergency medicine residents. Emergency medicine physicians experience nearly three times higher rates of career burnout than other physicians [18,19], and emergency medicine residents have demonstrated low levels of overall life satisfaction [20]. Wellness experts have called for a proactive, rather than reactionary, approach to improving the wellness of emergency medicine residents [21]. It is believed that physical activity is an inverse correlate of burnout among physicians, and engagement in physical activity is a modifiable behavior [11,22,23]. To date, there are no studies to our knowledge that have evaluated baseline physical activity among emergency medicine residents, and its effect on wellness is not described. Despite the perceived frenetic nature of the specialty of emergency medicine, the typical emergency medicine resident does not achieve the baseline physical activity recommendations posited by the USDHHS and the Centers for Disease Control and Prevention (CDC) [24,25] during a standard shift in the emergency department. When researchers placed pedometers on residents in a single, urban, academic, emergency medicine training program, only 9.9% of the residents took at least 10,000 steps during a shift [26]. Little is known about the physical activity behaviors in this population outside of the emergency department.

Pedometers have been shown to improve physical activity in different populations [27-29]. However, newer wearable devices for tracking physical activity have been used in an attempt to improve physical activity in specific populations. These wearable devices use complex proprietary algorithms to collect and provide physical activity data to the wearer, while being interconnected with computers and mobile phones. One study of internal medicine residents who used a Fitbit activity monitor in the clinical setting showed good adoption and adherence [12]. However, this study was not designed to measure the change in physical activity among residents after receiving the device, but rather the effect of the data provided by the device on their physical activity. Prior research has not shown how implementing a wearable exercise tracker will affect the physical activity behaviors of medical residents.

The primary purpose of this study was to measure the effectiveness of using a wearable device for tracking physical activity on the physical activity behaviors of emergency medicine residents. We hypothesized that self-reported physical activity levels would increase after receiving the device.

## Methods

### Study Design

This study was designed as a pre-post cohort study and involved both active data collection and participant-completed questionnaires. This study was approved by the institutional review board. All participants provided written informed consent and research was conducted in accordance with the Declaration of Helsinki. The data collection portion of this study lasted for 6 months, from September 1, 2014, to March 1, 2015.

The study population consisted of the members of a 3-year, accredited, academic, emergency medicine residency in the United States. The residency is composed of 62 total physicians, divided into 3 postgraduate years. Among the residents, 3 were involved as researchers and therefore were not eligible to participate. All other residents in the program were otherwise eligible to participate and all 62 residents were given a device.

### Outcome Measures

The primary outcome measure was the change in the self-reported days per week of at least 30 minutes of physical activity, measured by questionnaire at study start and after 1 month of physical activity tracker use.

The secondary outcome was the change in weekly physical activity—defined by the number of days per week with at least 10,000 steps or 30 minutes of active time—as measured by the Fitbit (FitBit Flex; FitBit Inc, San Francisco, CA, USA) wearable activity tracker compared with the baseline self-reported estimate of physical activity. The accuracy of wearable devices for tracking physical activity has been formally assessed and compared with the physical activity monitors in mobile phones [30]. The algorithm used by the Fitbit company products is proprietary; however, it has been previously used and validated in health services research [31,32]. The number of steps recorded by the Fitbit Ultra has been shown to correlate well with the ActiGraph activity monitor, a well-validated and frequently used exercise research tool [33]. The Fitbit device and step counting algorithm also appears to have good validity when compared with multiple other tools while walking in a controlled environment [30] but may underestimate physical activity under certain conditions such as cycling or other physical activity [34]. When applied to a population of cardiac rehabilitation patients, and compared with the ActiGraph research accelerometer as the gold standard, the same activity tracker used in this study was found to overestimate the amount of physical activity performed by participants [35].

Additional measures of interest included subjective characteristics specific to the adoption and continued use of the physical activity tracking device, measures of wellness, changes in physical activity behavior, and change in self-reported physical activity at 6 months. We conducted a stratified analysis of the population for the physical activity specific outcomes based on two predetermined factors: whether or not the participants continued to use their device throughout the entirety of the 6-month study period and whether or not the participants met the CDC recommended guidelines for adult physical activity at the start of the study, based on their self-reported physical activity in the baseline questionnaire. CDC guidelines for adults recommend “150 minutes of moderate-intensity aerobic exercise (ie, brisk walking) per week” [25].

### Study Protocol

All residents within this emergency medicine program were given a wearable physical activity tracker to improve their overall physical activity levels. Before receiving their device, the residents were asked if they would like to participate in this research study, advised that there would be no compensation offered to participate, and informed that receipt of the device would not be contingent on participation. At enrollment, all eligible participants were asked to complete a baseline questionnaire regarding demographic characteristics and physical activity habits (see baseline survey instrument in Multimedia Appendix 1). This questionnaire and all further questionnaires were conducted through SurveyMonkey software. Participants then received their devices and were asked to complete a 2-week acclimatization period before the initiation of electronic data collection. During this acclimatization period, participants were encouraged to wear and use the device. The purpose of this acclimatization period was to allow participants to activate their devices and learn how to use them in their regular daily life. Primary data collection from the devices occurred over the following month, September 2014. Participants were aware that their physical activity information would be collected during this period and were instructed to wear their devices as instructed by the device manufacturer on the packaging insert and on the manufacturer’s website. Specifically, participants were asked to wear their device at all times, with the exception of charging. The hospital training environment does not have stated restrictions on wristband or physical activity tracker use and the participants were able to wear the devices in the clinical setting. The choice to actively follow the physical activity data for 1 month, as opposed to a longer duration of time, was made by the investigators for several reasons. First, active data tracking time was limited to 1 month to minimize the impact of being a study participant on the daily lives of the emergency medicine residents. Second, there is a paucity of data on the length of time needed to create a lasting change in physical activity behavior among otherwise healthy physician volunteers with a physical activity tracker intervention.

The specific physical activity tracker used in this study allowed for near real-time physical activity information gathering and data downloads. Specifically, the device provides data on both steps and “active minutes” for each participant. “Active minutes” were calculated within the proprietary algorithm of the device; however, the device manufacturer describes “active minutes” as time measured when the device senses movement that correlates with physical activity above 3 metabolic equivalents (METs) for 10 consecutive minutes. This specific time cutoff was based on specific CDC guidelines for physical activity [25]. In order to facilitate regular data collection, all study participants were asked to create an account on the Fitbit Inc website and register their device for data tracking. Participants then shared access to their Web-based data for the duration of the study period. Data collection was conducted through a third-party application programming interface that pulled the physical activity data from the Fitbit.com servers and generated daily physical activity reports for each participant. These reports were collected for 1 month after which data from participants were only gathered to determine if they continued to use the device until the study period ended. Because prior research has shown that device-specific barriers, such as frequent charging, may decrease the number of days during follow-up that the participants can wear their device, active data tracking was limited to those days in which the participants wore their device for at least 100 steps. This limitation did not apply to the primary outcome measure.

Following the month of physical activity data gathering, participants were asked to complete a questionnaire assessing their use of the activity tracker, perceptions of the device, information on their physical activity during the past month, and a self-assessment of the impact of the device on their self-perceived physical activity and overall wellness. At 6 months, participants were asked to complete a final follow-up survey to assess their use and perceptions of the device as well as their current physical activity levels.

### Statistical Analysis

Data analysis included descriptive statistics of demographic characteristics, measures of wellness, physical activity, and perceptions about the wearable activity tracker. As the data did not satisfy the assumption of normality, statistical comparisons were conducted with the nonparametric Wilcoxon signed rank test, for the primary and secondary outcomes of interest as well as for the stratified analyses within these outcomes. Statistical significance was defined as a *P* value of <.05. Given the small population size of this pilot study and lack of research precedent for this type of intervention, no power calculation was conducted. All statistical analyses were conducted in SAS version 9.4 (SAS Institute Inc).

## Results

### Study Population

**Figure 1:**
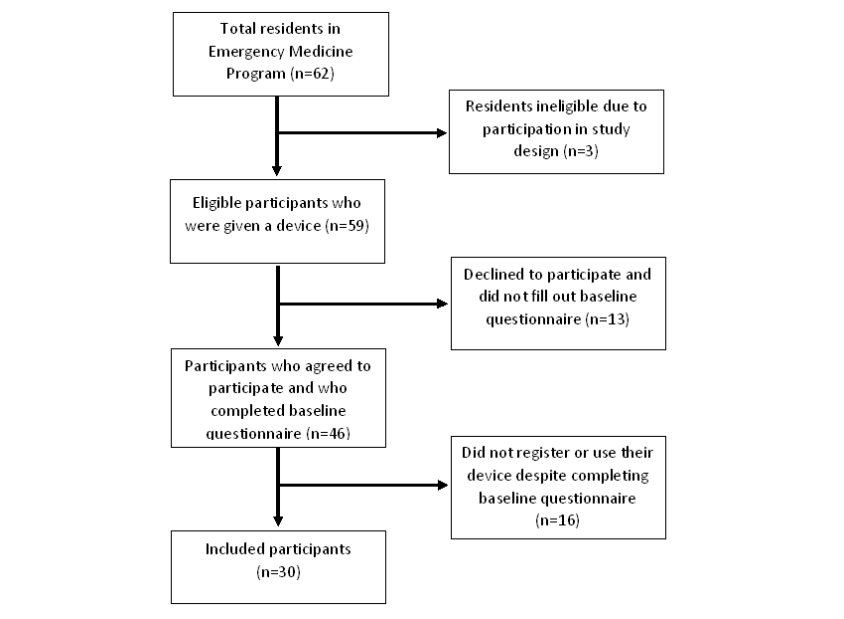
Flow diagram of study inclusion.

Of the 59 eligible residents, 30 ultimately participated in the active data tracking portion of this study, where they used the physical activity tracker for at least 1 week during the first month of follow-up and completed 3 questionnaires over the 6-month period. Of the 59 residents who received a device before the start of the study, 46 (78%) were initially willing to participate and completed the baseline questionnaire, but of these participants, 16 (35%, 16/46) did not register or wear their devices and were excluded from the study analysis (Figure 1). The participants who were excluded were similar in demographic characteristics and baseline physical activity behaviors to the study population based on responses to the initial questionnaire.

Among the 30 study participants, the median age was 28 years (interquartile range, IQR, 4.0), approximately half (53%, 16/30) were male, 40% (12/30) were married, and 10% (3/30) had children. In addition, 3 participants (10%, 3/30) had and were still using a physical activity tracker at the start of the study and 1 participant previously had a device but had stopped using it before the start of the study. The overall perception of physical activity trackers at baseline was positive, with 26 (87%, 26/30) participants describing devices as helpful or possibly helpful on a 5-point Likert scale. The participants generally described themselves as moderately healthy (median 2.0, IQR 2.0, on a scale of 0-4 ranging from not at all healthy to extremely healthy; Table 1).

Despite rating exercise as personally “important” (median 3.0, IQR 1.0) on a 5-point scale ranging from not at all important (score of 0) to extremely important (score 4), participants felt that they exercised less than they would like. The median number of different types of physical activities reported by the cohort was 2.0 (IQR 1.0). With regard to how work influenced their physical activity behaviors, the majority, 23/ (77%, 23/30), felt that residency training and their work schedule negatively affected their physical activity behaviors. Nearly everyone in the study, that is, 29 of 30 participants (97%), described physical activity in general as having a positive impact on their wellness, and all study participants felt that an increase in physical activity levels would have a positive impact on their wellness (Table 1).

**Table 1:** Demographic characteristics, physical activity, and wellness perceptions among study participants.

Study variables		Participated in Fitbit tracking (N=30)
Age in years, median (IQR^a^)		28 (4.0)
Sex, male, n (%)		16 (53)
Relationship status, single, n (%)		12 (40)
With children younger than 18 years, n (%)		3 (10)
**Do you have a Fitbit or other exercise tracker and if yes, do you still use it? n (%)**		
	Yes, still use	3 (10)
	Yes, no longer use	1 (3)
	No	26 (87)
**What is your perception of biometric monitoring or other exercise tracking devices, such as the Fitbit? n (%)**		
	Not helpful, possibly harmful	0 (0)
	Possibly not helpful	1 (3)
	No opinion	3 (10)
	Possibly be helpful	24 (80)
	They are helpful	2 (7)
Personal health perception, scale 0-4, median (IQR) (0=not at all healthy, 2=moderately healthy, 4=extremely healthy)		2.0 (2.0)
How important is exercise to you? Scale 0-4, median (IQR) (0=not at all important, 2=moderately important, 4=extremely important)		3.0 (1.0)
How much physical activity or exercise do you get? Scale 0-4, median (IQR) (0=much too little, 2=about the right amount, 4=too much exercise)		1.0 (2.0)
How many different physical activities or exercises do you participate in? Median (IQR)		2.0 (1.0)
**How do you feel your work schedule impacts your physical activity? n (%)**		
	Negative impact	23 (77)
	No impact	6 (20)
	Positive impact	1 (3)
**How do you feel residency training has impacted your physical activity? n (%)**		
	Negative impact	23 (77)
	No impact	4 (13)
	Positive impact	3 (10)
**Do you feel that a typical ED^b^ shift provides you with sufficient physical activity for the day? n (%)**		
	No	23 (77)
	Not sure	2 (7)
	Yes	5 (16)
**How does working an overnight shift impact your physical activity? n (%)**		
	Negative impact	26 (87)
	No impact	3 (10)
	Positive impact	1 (3)
**How does physical activity affect your wellness? n (%)**		
	Negative impact	0 (0)
	No impact	1 (3)
	Positive impact	29 (97)
**How would an increase to your physical activity affect your overall wellness? n (%)**		
	Negative impact	0 (0)
	No impact	0 (0)
	Positive impact	30 (100)

^a^IQR: interquartile range.

^b^ED: emergency department.

### Outcome Measures

The primary outcome measurement, change in self-reported number of days of physical activity per week after 1 month of device use, was not statistically significantly different from the baseline self-reported number of days of physical activity. The median self-reported number of days of exercise per week before receiving the device was 2.5 (IQR 1.9) and after 1 month was 2.8 days (IQR 1.5, *P*=.36; Table 2).

The stratified analysis of the primary outcome showed that among those participants with physical activity below the CDC recommended amount of weekly physical activity at baseline, there was a statistically significant increase in the number of weekly days of physical activity from 1.5 (IQR 0.9) to 2.4 (IQR 1.2), *P*=.04, at 1 month, and an increase from baseline to 2.0 (IQR 2.0) days per week at 6 months (*P*=.04). The population of participants who met or exceeded the CDC recommended guidelines for physical activity at study start did not have a statistically significant change in their physical activity at 1 month (*P*=.69; Table 2). Among participants who continued to use their device at 6 months (10/30, 33%), there was no statistically significant change in physical activity from their baseline at study start. The same was true of people who stopped using the device before the end of the study period (20/30, 67%; Table 2).

The secondary outcome of interest, change in days per week of physical activity as measured by the physical activity tracker compared with self-reported baseline days per week of physical activity did not reveal a statistically significant change in physical activity. The median number of days of physical activity as measured by the device was 2.5 (IQR 2.7) compared with the baseline median number of days of exercise per week of 2.5 (IQR 1.9). The median number of eligible days recorded by the device where the participant recorded at least 100 steps was 27.5 (IQR 8) over the course of the 30-day month. There was no statistically significant difference in physical activity levels at 1 month among those who met or did not meet CDC recommended exercise guidelines (*P*=.69). Nor was there a statistically significant difference among those who continued to use the device for the entirety of the study period when measured at 1 month compared with themselves (*P*=.85), or among the group of people who discontinued use before 6 months (*P*=.34; Table 2).

### Continued Use

Barriers to the continued use of the wearable physical activity tracker were addressed in both the 1-month and 6-month follow-up questionnaires. When study participants were asked to list the barriers to continued use of their physical activity tracker at 1 month, half listed forgetfulness—either forgetting to charge or forgetting to wear—the device. However, the other half of participants did not note any barriers to continued use. Barriers to continued use are listed in Table 3 and include the following: not wanting to wear the device, boredom, the belief that the device was not accurately measuring physical activity, and that it was not increasing overall physical activity. Fashion and the device breaking were also noted as barriers.

**Figure 2:**
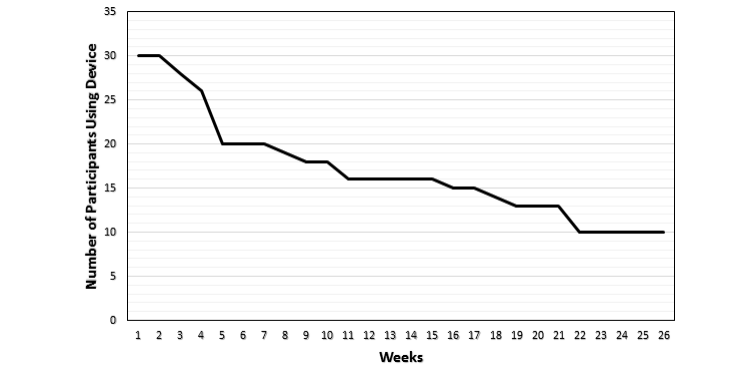
Number of participants, by week, who continued to use their wearable device for tracking physical activity during follow-up.

At 1 month, 18 of 30 (60%) participants described a positive impact on their wellness because of physical activity tracker use and 16 of 30 (53%) listed physical activity tracker use as having a positive impact on their physical activity. Of the 30 participants, 20 participants (67%) continued to use their device after 1 month, but only 10 (33%) participants still used their device after 6 months (Table 3). Figure 2 describes in graphical format the number of study participants who continued to use their device, by week, during the 6-month follow-up period.

Among those who stopped using the device by 6 months (20 of 30 participants), the participants listed both subjective and functional device issues as their principal reason for stopping use of the device, which were similar to the reasons for discontinued use at 1 month. Reasons given for discontinued use included the following: the impression that the device was no longer changing their exercise habits, boredom with the device, the impression that it was not accurately recording physical activity, and the impression that the device was a fad. Device-specific reasons for discontinued use at 6 months included loss of the device, wristband breaking, and issues with charging the device frequently (Table 3).

Among participants who continued to use the device for the entire study period (10 of 30), 4 of 10 participants (40%) listed liking the data provided by the device as their reason for continued use. Additionally, 3 of 10 participants (30%) found that the device reminded them to exercise. And 2 of 10 participants (20%) listed peer pressure as their principal reason for continued use. One person listed the device making him or her feel more physically fit as the main reason for continued use (Table 3).

**Table 2:** Self-reported physical activity among study participants at baseline, 1 month, and 6 months stratified by continued use and by level of physical activity before receiving device.

	Estimate of the number of days exercised per week at baseline before receipt of physical activity tracker (n=30)	Estimate of the number of days exercised per week after 1 month of physical activity tracker use (n=30)	Physical activity tracker measured number of days per week of exercise at 1 month of use (n=30)	Estimate of the number of days of exercise per week 6 months after receipt of physical activity tracker (n=30)
Study population (n=30), median (IQR^a^)	2.5 (1.9)	2.8 (1.5) *P*=.67	2.5 (2.7) *P*=.69	3.0 (2.0) *P*=.36
Met CDC^b^ recommendations for adult physical activity prior to study start (n=20), median (IQR)	3.4 (0.9)	2.9 (1.8) *P*=.52	2.8 (2.8) *P*=.27	3.5 (2.5) *P*=.69
CDC recommendations for adult physical activity prior to study start not met (n=10), median (IQR)	1.5 (0.9)	2.4 (1.2)^c^ *P*=.04	2.0 (1.7) *P*=.39	2.0 (2.0)^c^ *P*=.04
Continued to use device for 6-month study period (n=10), median (IQR)	2.5 (1.9)	2.7 (0.9) *P*=.97	1.9 (2.6) *P*=.39	3.0 (2.0) *P*=.85
Discontinued physical activity tracker use prior to study end (n=20), median (IQR)	2.5 (1.9)	2.9 (2.0) *P*=.64	2.6 (2.7) *P*=.86	3.0 (2.0) *P*=.36

^a^IQR: interquartile range.

^b^CDC: Centers for Disease Control and Prevention.

^c^Significant at *P*<.05 level, Wilcoxon signed rank test.

**Table 3:** Follow-up questionnaires on the use and barriers to use of the wearable device for tracking physical activity at 1 month and 6 months.

Questionnaire responses at 1 month and 6 months, n=30		n (%)
**Barriers to use of the physical activity tracker at 1 month (cumulative percentage)^a^**		
	No barriers noted	15 (50)
	There were days that I forgot to charge it	15 (50)
	There were days that I forgot to wear it	15 (50)
	It was not increasing my physical activity	4 (13)
	I did not like wearing it on my wrist	4 (13)
	I became bored with it	4 (13)
	It was not accurately measuring my physical activity	4 (13)
	Fashion	3 (10)
	It broke or stopped working	3 (10)
	I felt like I could not be physically active	2 (7)
	I became injured	1 (3)
	I lost the device	1 (3)
Physical activity tracker use had a positive impact on personal wellness at 1 month		18 (60)
Physical activity tracker use had a positive impact on physical activity at 1 month		16 (53)
Continued to use the physical activity tracker at 1 month		20 (67)
Continued to use the physical activity tracker at 6 months		10 (33)
**Principal reason for stopping use of the physical activity tracker by 6 months (n=20)**		
	The device was not changing my exercise habits	3 (15)
	The device broke	3 (15)
	I became bored with the device	2 (10)
	The device was not accurately recording my physical activity	2 (10)
	I lost the device	2 (10)
	I found the device to be uncomfortable	2 (10)
	The wristband broke and I did not replace it	2 (10)
	I did not want to wear the device on my wrist	1 (5)
	The device would not charge	1 (5)
	The device is a fad	1 (5)
	I encountered issues with charging the device frequently	1 (5)
**Principal reason for continued use of the physical activity tracker at 6 months (n=10)**		
	I liked the data provided by the device	4 (40)
	The device reminds me to exercise	3 (30)
	Peer pressure from other people wearing the device	2 (20)
	The device makes me feel more physically fit	1 (10)

^a^Multiple answers eligible.

## Discussion

### Principal Findings

The primary objective of this study was to examine the effectiveness of a wearable device for tracking physical activity on self-reported levels of physical activity among a relatively healthy group of emergency medicine residents 1 month after receiving a physical activity tracker. Within this cohort of 30 emergency medicine residents, there was no overall statistically significant change in self-reported average number of days of physical activity per week 1 month after receiving the physical activity tracker. However, within the prespecified subgroup of residents who did not meet the CDC recommended minimum level of physical activity before receiving the device, there was a statistically significant increase in self-reported weekly physical activity from baseline (1.5 days) to 1 month (2.4 days) and 6 months (2.0 days). Despite a lack of measurable change in the primary end point, the majority of study participants felt that receiving and using the physical activity tracker had a positive impact on their physical activity levels and overall wellness. The broad implications of these findings suggest that these devices do not appear to have a negative impact on physical activity, may be beneficial within specific populations, and may improve wellness in ways that are not measurable with self-reported or device-provided data. These findings may help other emergency medicine or medical training programs implement physical activity programs for residents to improve their wellness by targeting interventions to those who are not physically active and by pairing a physical activity tracker intervention with additional behavioral interventions.

There are several potential explanations for why we did not observe a substantial effect of the physical activity tracker on physical activity levels after 1 month for our entire study population. First, the population in our study was young, physically active at enrollment, and presumably healthy, with two-thirds of participants already meeting CDC guidelines for weekly exercise. Thus, the potential effect of the physical activity tracker among an already active population is likely smaller and may require a larger study to find a statistically significant increase in physical activity. This is supported by our finding that the physical activity tracker was only significantly effective among the subgroup of participants who had not met CDC guidelines for exercise at baseline. Another potential explanation for our findings was that a physical activity tracker alone was not enough to encourage a major change in physical activity. Our study did not use a specific external behavioral change technique, such as a study coordinator helping the participants set an exercise goal. Instead, participants had the opportunity to choose to use the device and its built-in tools as a motivator. Nonetheless, the physical activity tracker used in this study, when paired with the website and mobile phone app, uses many behavior change techniques that have been previously described in the literature, including goal-setting behavior, feedback on behavior, social comparison, prompts and cues, social and other nonspecific rewards, and immediate feedback [36]. Finally, one-third of study participants discontinued use of the physical activity tracker before the 1-month period, which may have reduced the potential effectiveness of the device.

### Wellness and Physical Activity

Nonetheless, this population of emergency medicine residents, while generally healthy, is still at risk for psychosocial problems such as career burnout and lack of wellness [19-22]. Even emergency medicine residents who described themselves as moderately healthy at study enrollment felt that they nonetheless exercised less than they would like, suggesting that before using their physical activity tracker the participants in this cohort were both aware of their own levels of physical activity and placed a value on their own wellness and the effect that physical activity has on it. Study participants described physical activity as personally important and felt that an increase in their physical activity would improve their overall wellness. Residency training, work schedule, and night shifts were all listed as having a negative impact on their physical activity levels, suggesting that physical activity tracker or other interventions to improve physical activity and resident wellness are important.

### Barriers to Adoption and Continued Use

Evidence does suggest that a physical activity tracker may increase physical activity; however, barriers to adoption and continued use may limit the overall effectiveness. In a qualitative analysis of the Pedometer and consultation-UP trial (PACE-UP), which used pedometers and notebooks for participants and nurse follow-up as their intervention, the authors found the process of monitored physical activity to be beneficial to most participants with the caveat that some participants perceived barriers when the equipment failed to accurately record their activity [37]. This mistrust of monitoring devices was also shown in our results, specifically among those who discontinued use of the physical activity tracker. This specific characteristic of physical activity trackers is a barrier that must be addressed in future research. It is difficult to measure the effect that even a single episode of unmeasured or incorrectly measured physical activity might have on adherence, but it has the potential to bias results. Nonetheless, the stratified analysis of participants who either continued to use their physical activity tracker throughout our study or who stopped during the study period yielded no overall change in measured or self-reported physical activity.

With two-thirds of the participants discontinuing use of the physical activity tracker at 6 months, a consideration of the reasons for discontinuation is warranted to help inform future studies that may assess a physical activity tracker intervention among a healthy population. Reasons for discontinued use were varied but broadly included subjective reasons such as not wanting to wear the device on the wrist, the belief that the device was not accurately recording physical activity, and device-specific reasons such as malfunction, loss, comfort, and fashion. In prior research among an internal medicine resident population, compliance and adherence to interventions with an older generation physical activity tracker were better when paired with an ongoing exercise program and with weekly reminder emails [12]; however, we chose not to add these elements to our research protocol in an attempt to focus on the device-specific benefit and create an intervention that would be simple, reproducible, and scalable. Future research on the use of a physical activity tracker for health and wellness promotion will likely continue to be hindered by these elements. However, researchers who choose to use the physical activity tracker for health promotion may see an improvement in continued use among participants who appreciate the data provided by the device and the reminder to exercise that the physical presence of the device on the arm provides. Additionally, using the data provided by this type of device appears to be somewhat limited by the user.

The physical activity tracker used in this study was specifically designed to capture ambulatory activities; however, the company allows for inputting the duration of alternative physical activities such as swimming, cycling, weight lifting, and yoga into the computer and application interface. We did not specifically ask our study participants to input or record nonambulatory activities. This likely would have primarily affected only the secondary outcome of this study, which was device-measured active days. However, had the participants logged their nonambulatory activities, this would have been captured as active time. We did not differentiate between personally logged and device-measured activities. Nonetheless, in the initial survey we screened participants for their preferred physical activities, and the median number of different activities was 2.0 (IQR 1.0). One additional potential reason why participants discontinued use of the physical activity tracker was the limited ability of the physical activity tracker to record accurate and complete information about a participant’s physical activity. All participants endorsed performing physical activities that are readily captured by the device, such as walking, running, jogging, or hiking. We did not capture their primary mode of physical activity, and there is therefore the possibility of bias in the effectiveness of the device and the primary outcome, should the participants feel as though their physical activity was not being measured correctly. A total of 2 of the 20 participants who eventually stopped using the device noted that the device was not measuring their physical activity correctly, although it is unclear if this was specific to failure of the device to record nonambulatory physical activities or mismeasurement of activities that the device is supposed to accurately capture, such as walking. Other studies have also reported similar barriers to using these devices, including the “novelty effect” wherein continued use declined, lack of adherence among participants, and technical issues with the device or website [34]. Nonetheless, Fitbit devices have been used in studies of cardiac rehabilitation programs with better overall adherence to use [38], and have shown promise for physical activity interventions among obese sedentary adult women [39], and for patients with chronic obstructive pulmonary disease [40]. These findings point to a possible enhanced benefit among less physically active users, which is also suggested by our results. The overall effectiveness of the device among a less healthy study population may be influenced by multiple factors including regular contact with medical professionals and the variety of non–device-specific behavioral modification techniques used in their research protocols—such as a nurse or study coordinator helping to set goals.

### Limitations and Future Directions

Several limitations were identified in this study. This was a single institutional study, albeit a large and diverse residency program. Study data suggest that the baseline physical activity levels were higher than that described in other studies of resident physical activity. The residency leadership’s emphasis on well-being and exercise, as demonstrated by the gift of a physical activity tracker, may have biased resident participation, and participants may have been more likely to overreport physical activity or even use the physical activity tracker more than they would normally have had it not been a gift from their employer. Of the study investigators, 3 were emergency medicine trainees during enrollment and data acquisition, and although this poses a potential source of bias in that the study participants frequently interacted with the investigators, implementing this type of intervention in the future will most likely also involve peer-to-peer interaction. It is unclear how this type of interaction can bias the results of this type of study, but it most likely encouraged participants to exercise more frequently and possibly could have led to overreporting of physical activity. The small sample size also limited our ability to conduct subgroup analyses, and future research may be needed to examine the effect of a physical activity tracker among people of different demographic groups.

This study is subject to selection bias. Slightly more than half of the eligible participants in the emergency medicine residency were part of the active data collection and follow-up. However, the 16 residents who initially enrolled in the study and completed the baseline questionnaire, but did not participate in further active data collection, had similar baseline characteristics and self-reported levels of physical activity. This study also involved 3 participant questionnaires and therefore suffers from the inherent biases of research with cross-sectional elements. To decrease the amount of recall bias, subjective recall periods were kept intentionally short and specific. Furthermore, participants were aware that they would be providing estimates of their physical activity habits before being asked for them and were thus more likely to accurately recall and report these values. Conversely, this study involved a physical activity intervention, which could have caused unintentional inflation of self-reported exercise frequency. To mitigate this possible source of bias, the physical activity data from the device itself were used in addition to the self-reported amount of physical activity from the participants, and results did show high agreement. It is also possible that the physical activity data provided by the website and mobile app associated with this physical activity tracker could have influenced the self-reported amount of physical activity at 1 month. It is unclear if this potential bias could have masked the effect of the intervention. Further research must be performed to determine the degree to which access to a person’s physical activity data can influence that person’s self-reported physical activity. It also must be noted that the optimal time period during which to observe a sustained change in physical activity for this type of intervention is unknown. The follow-up time of 1 month may have been too short for our primary outcome. Our choice to limit active follow-up to 1 month was made for several reasons. First, as this was a pilot study, we did not want to unduly burden the study participants as they are medical residents with significant demands on their time and they were asked to regularly interface with the mobile app or website and use the device. Second, the only other study of an intervention using a physical activity monitor on a similar population [10] chose a 6-week by 6-week time period as an appropriate length of time for its crossover randomized clinical trial. Our study allowed for a 2-week acclimatization period, followed by 1 month of active monitoring. Our study specifically aimed to address feasibility and effectiveness of the Fitbit device over a short time period. Our primary focus was not on maintenance of the health behavior; however, this will be of paramount interest for future investigators who wish to use a physical activity tracker in a similar population. Finally, this study did not use validated physical activity or wellness tools, and thus caution should be used when interpreting these data. Future studies should seek to use validated instruments for their study population to increase the ability to compare results across study populations.

Additional limitations about the physical activity tracker used in this study should be noted. First, the device, even when worn correctly, may have underrepresented [34] or overrepresented [35] the amount of physical activity performed by each participant—a known problem that has previously been described in the literature. Second, the device itself required the user to remember to use it and to keep it charged, both of which allowed for inconsistencies in the number of days eligible for active data tracking. Nonetheless, daily use of the device was generally good and the number of physically active days per week as recorded by the device was similar to, if only slightly lower than, the median number of active days provided on the 1-month questionnaire. Finally, the study was limited with respect to determining the true amount of physical activity performed by each participant during follow-up. The apparent lack of difference between the device-measured and self-reported physical activity observed in this study must be viewed in light of the small sample size. It remains unclear how behavioral change should be measured, either with a questionnaire or with the data provided by the device, when using a physical activity tracker as an intervention. We hope that future research in this area can address the limitations largely due to the relatively small sample size of our pilot study. We are encouraged by portions of the results that suggest an improvement in overall wellness and physical activity within the subset of the population. We suggest that future research address some of the device-specific and adherence concerns voiced by our participants. Research that has paired these devices with behavioral interventions has also shown promise and should be explored in a larger sample of healthy participants as well. We also suggest lengthening the overall study duration to more accurately capture adherence to behavior change.

### Conclusions

The implementation of a physical activity tracker among a healthy population of emergency medicine residents did not change the overall self-reported physical activity at 1 month and 6 months. However, there was a significant improvement in the amount of physical activity among the residents with preintervention physical activity levels below the CDC recommended guidelines. Subjective improvements in overall wellness and physical activity were noted among the whole study population. Adherence waned over the study period with only one-third of participants continuing to use the device at 6 months. Our pilot study findings may provide helpful information for residency programs that may be contemplating a wearable physical activity tracker intervention among their residents or others who may be considering a similar intervention among a relatively healthy population of adult participants.

## Appendix

Multimedia Appendix 1 Baseline survey instrument.

## Abbreviations

CDC: Centers for Disease Control and Prevention
IQR: interquartile range
USDHHS: US Department of Health and Human Services
WDTPA: wearable device for tracking physical activity
