# Supplementary material for: Assessing the Influence of a Fitbit Physical Activity Monitor on the Exercise Practices of Emergency Medicine Residents: A Pilot Study
Source: JMIR Mhealth Uhealth. 2017 Jan 31;5(1):e2. doi: 10.2196/mhealth.6239 (PMC5309436; doi:10.2196/mhealth.6239)
Supplement: Multimedia Appendix 1 [file mhealth_v5i1e2_app1.pdf]

Pilot Study to Assess the Influence of a Fitbit Physical Activity Monitor on the Exercise Practices of Emergency Medicine Residents

Baseline Survey:

1) What is your age? (number)
2) Are you a male or female? (male/female)
3) Which race/ethnicity best describes you?
    a. American Indian or Alaskan Native
    b. Asian/Pacific Islander
    c. Black or African American
    d. Hispanic American
    e. White/Caucasian
    f. Multiple: (please specify)
4) Which of the following best describes your current relationship status?
    a. Married
    b. Widowed
    c. Divorced
    d. Separated
    e. In a domestic partnership or civil union
    f. Single, but cohabitating with a significant other
    g. Single, never married and not cohabitating
    h. Other (please specify)
5) Do you have children under the age of 18 yo? (yes/no)
6) Do you have experience using a Fitbit device or other physical activity monitoring devices?
    a. Yes, and I use one currently
    b. Yes, but I no longer use one
    c. No
    d. I do not know what a physical activity monitoring device is
7) Do you plan to activate your Fitbit device and use it while working in the ED? (yes/no/maybe)
8) What is your perception of biometric monitoring devices such as the Fitbit with regard to their affect on physical activity?
    a. They are helpful
    b. They might be helpful
    c. No opinion
    d. They might be harmful
    e. They are harmful
9) How physically healthy are you?
    a. Extremely healthy
    b. Very healthy
    c. Moderately healthy
    d. Slightly healthy
    e. Not at all healthy
10) How important is exercise to you?
    a. Extremely important
    b. Very important
    c. Moderately important
    d. Slightly important

   e. Not at all important

11) What is your perception of the amount of exercise you get?
    a. Much too much
    b. Somewhat too much
    c. Slightly too much
    d. About the right amount
    e. Slightly too little
    f. Somewhat too little
    g. Much too little
12) What type of physical activity or exercise do you do? (select all that apply)
    a. Lift weights
    b. Walk
    c. Run
    d. Hike
    e. Swim
    f. Dance
    g. Aerobics
    h. Pilates
    i. Play a team sport
    j. Other (please specify)
13) Please estimate how many <u>days per week</u>, on average over the past month, you participated in physical activity for at least 30 minutes (number)
14) How do you feel your work schedule impacts your physical activity?
    a. My work schedule negatively impacts my physical activity
    b. My work schedule does not affect my physical activity
    c. My work schedule positively affects my physical activity
15) How do you feel residency training impacts your physical activity?
    a. Residency training negatively impacts my physical activity
    b. Residency training does not affect my physical activity
    c. Residency training positively affects my physical activity
16) Do you feel as though a typical shift in the ED provides you with sufficient physical activity for the day?
    a. Yes
    b. No
    c. Unsure
17) How does working overnight shifts in the ED impact your physical activity?
    a. Overnight shifts do not affect my physical activity
    b. Overnight shifts negatively affect my physical activity
    c. Overnight shifts positively affect my physical activity
18) How does exercise affect your overall wellness?
    a. It does not affect my wellness
    b. It has a positive effect on my wellness
    c. It has a negative effect on my wellness
19) How do you feel that increased physical activity would affect your overall wellness?
    a. Increased physical activity would improve my wellness
    b. Increased physical activity would worsen my wellness
    c. Increased physical activity would not change my wellness
20) How do you feel the Fitbit will influence your physical activity? (free text)
21) Do you have any thoughts or concerns about the Fitbit that were not addressed in this survey? (free text)
